# Characterization on Lead-Free Hybrid Perovskite [NH_3_(CH_2_)_5_NH_3_]CuCl_4_: Thermodynamic Properties and Molecular Dynamics

**DOI:** 10.3390/molecules27144546

**Published:** 2022-07-16

**Authors:** Ae Ran Lim, Sang Hyeon Park

**Affiliations:** 1 Graduate School of Carbon Convergence Engineering, Jeonju University, Jeonju 55069, Korea; klly89@naver.com; 2 Department of Science Education, Jeonju University, Jeonju 55069, Korea

**Keywords:** organic-inorganic hybrid, perovskite, ferroelasticity, nuclear magnetic resonance, thermodynamic properties

## Abstract

It is essential to develop novel zero- and two-dimensional hybrid perovskites to facilitate the development of eco-friendly solar cells. In this study, we investigated the structure and dynamics of [NH_3_(CH_2_)_5_NH_3_]CuCl_4_ via various characterization techniques. Nuclear magnetic resonance (NMR) results indicated that the crystallographic environments of ^1^H in NH_3_ and ^13^C on C3, located close to NH_3_ at both ends of the cation, were changed, indicating a large structural change of CuCl_6_ connected to N–H···Cl. The thermal properties and structural dynamics of the [NH_3_(CH_2_)*_n_*NH_3_] cation in [NH_3_(CH_2_)*_n_*NH_3_]CuCl_4_ (*n* = 2, 3, 4, and 5) crystals were compared using thermogravimetric analysis (TGA) and NMR results for the methylene chain. The ^1^H and ^13^C spin-lattice relaxation times (T_1ρ_) exhibited similar trends upon the variation of the methylene chain length, with *n* = 2 exhibiting shorter T_1ρ_ values than *n* = 3, 4, and 5. The difference in T_1ρ_ values was related to the length of the cation, and the shorter chain length (*n* = 2) exhibited a shorter T_1ρ_ owing to the one closest to the paramagnetic Cu^2+^ ions.

## 1. Introduction

Recently, research on solar cells based on organic-inorganic hybrid materials has progressed very rapidly [1,2,3,4]. Initially, CH_3_NH_3_PbX_3_ (X = Cl, Br, I)-based thin-film photovoltaic devices were used as solar cells. Despite the development of CH_3_NH_3_PbX_3_ as a hybrid solar cell, it readily decomposes in humid air, and Pb toxicity is a major concern [5,6,7]. Therefore, its replacement with environment-friendly hybrid perovskite solar cells is vital.

Further, novel groups of perovskite materials, such as [(CH_3_)_2_NH_2_]Zn(HCOO)_3_, consisting of an organic cation and a metal ion, have been discussed [8,9,10,11,12,13,14]. They exhibited potential for application in memory manipulation devices and next-generation memory storage technology. In addition, it is necessary to study the structure and dynamics of new materials with zero- and two-dimensional (2D) hybrid perovskites, such as eco-friendly [NH_3_(CH_2_)_*n*_NH_3_]*M*X_4_. The organic-inorganic hybrid [NH_3_(CH_2_)*_*n*_*NH_3_]*M*X_4_ (*n* = 2, 3, 4, ···), where M is a divalent metal ion, and X is a halide ion, crystallizes in perovskite-type layered structures [15,16,17,18,19,20,21,22,23,24,25,26,27,28,29,30,31]. The organic [NH_3_(CH_2_)_*n*_NH_3_] cation of the hybrid complex influences properties, such as structural flexibility and optical characteristics, whereas the inorganic (*M*X_4_ or *M*X_6_) anions affect the thermal and mechanical properties [32,33]. For *M* = Mn, Cu, or Cd, the structure consists of the corner shared octahedral (*M*X_6_)^2−^ alternated with organic layers and is 2-dimensional, while for *M* = Co or Zn, the structures are tetrahedral (*M*X_4_)^2−^ sandwiched between layers of organic cations and 0-dimensional. The ammonium ions at the organic-cation terminals form N−H∙∙∙X hydrogen bonds with halide ions of the metallic inorganic layer [34,35,36]. For long chains, in complexes where *n* is 5 or more, structural changes due to conformational changes of the chains are important [37]. Among them, an interesting group of hybrid materials is perovskite-type layered [NH_3_(CH_2_)_5_NH_3_]CuCl_4_. Its crystal structure consists of 2D inorganic CuCl_4_ layers and 1,5-diaminopentane cations. The [NH_3_(CH_2_)_5_NH_3_] organic chains exhibit the longest c-axis. [NH_3_(CH_2_)_5_NH_3_]CuCl_4_ crystallizes in the monoclinic space group *P2_1_/c*, with unit-cell parameters of a = 7.747 Å, b = 7.203 Å, c = 21.512 Å, Z = 4, and β = 98.48° [38].

Filloleau et al. [39] and Kanel et al. [40] reported the magnetic, optical properties, and electron paramagnetic resonance studies of [NH_3_(CH_2_)_5_NH_3_]CuCl_4_ crystals. Recently, the thermal properties and structural molecular dynamics of [NH_3_(CH_2_)*_n_*NH_3_]CuCl_4_ (*n* = 2, 3, and 4) crystals containing Cu^2+^ ions have been reported [41,42,43]. However, a detailed report on the [NH_3_(CH_2_)_5_NH_3_]CuCl_4_ crystal is yet to be published. A lot of research has been done on the electric and conductive properties of this type of compound [44,45,46,47].

In this study, the crystal structure, thermodynamics, and ferroelasticity of [NH_3_(CH_2_)_5_NH_3_]CuCl_4_ were studied to investigate the CuCl_6_ anion, which is responsible for the thermal and mechanical properties. Additionally, to obtain information on the coordination geometry and molecular dynamics of the [NH_3_(CH_2_)_5_NH_3_] cation, nuclear magnetic resonance (NMR) chemical shifts and spin-lattice relaxation times (T_1ρ_) for ^1^H and ^13^C were measured using the magic angle spinning (MAS) method. The variations in physicochemical properties of this crystal according to the temperature change were explained by considering the cation and the CuCl_4_ anion. The influence of the CH_2_-group length in the [NH_3_(CH_2_)*_n_*NH_3_] cation of [NH_3_(CH_2_)*_n_*NH_3_]CuCl_4_ (*n* = 2, 3, 4, and 5) has also been discussed with reference to a previous report. These results, which consider the methylene chain length, could be useful for facilitating diverse environment-friendly applications in the future.

## 2. Results

### 2.1. Crystal Structure

**Figure 1:**
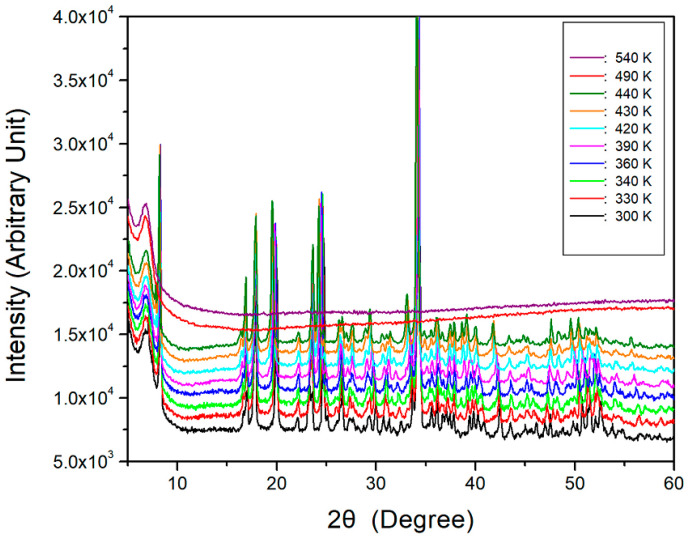
XRD powder patterns of the [NH_3_(CH_2_)_5_NH_3_]CuCl_4_ crystal at different temperatures.

The X-ray diffraction (XRD) powder patterns of the [NH_3_(CH_2_)_5_NH_3_]CuCl_4_ crystal were obtained at different temperatures during heating, and the results are shown in Figure 1. The XRD patterns from 300 K to 440 K were identical, and the XRD patterns at temperatures above 440 K were due to the melting of the crystal. Additionally, the crystal structure is monoclinic, and the lattice constants, analyzed from the single-crystal XRD results, were a = 7.7385 Å, b = 7.2010 Å, c = 21.5308 Å, β = 98.493°, and Z = 4, with the space group *P2_1_/c*. This result is consistent with a previous report [38].

### 2.2. Thermal Property and Ferroelastic Twin Domain

**Figure 2:**
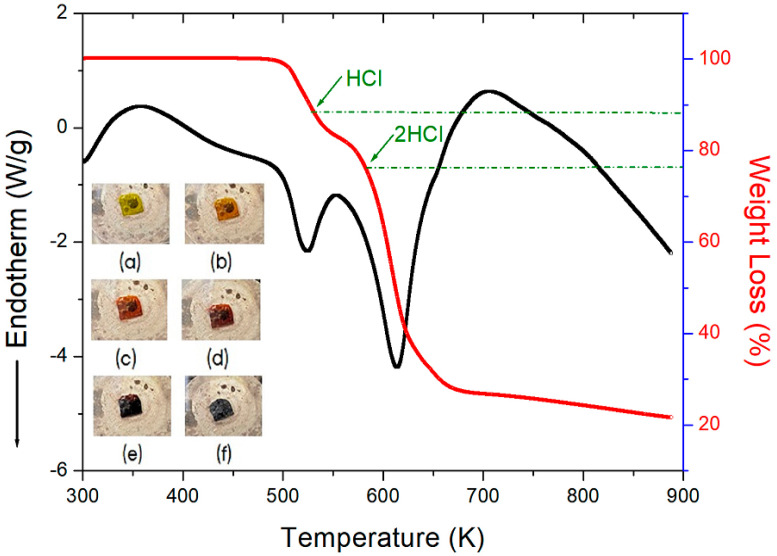
TGA and DTA curves of [NH_3_(CH_2_)_5_NH_3_]CuCl_4_ (inset: changes in the crystal at the following temperatures: (**a**) 300 K, (**b**) 330 K, (**c**) 390 K, (**d**) 430 K, (**e**) 490 K, and (**f**) 540 K).

To understand the thermodynamic properties, thermogravimetric analysis (TGA) and differential thermal analysis (DTA) results measured at a heating rate of 10 K/min are shown in Figure 2. The first occurrence of molecular weight loss, indicating the initiation of partial thermal decomposition, occurred at approximately 514 K. As the temperature increased, the molecular weight of the [NH_3_(CH_2_)_5_NH_3_]CuCl_4_ crystal decreased. TGA results of a similar compound were reported by another group previously [24,27,48]. The 12% and 24% losses, calculated from the total molecular weight, were caused by the decomposition of HCl and 2HCl, respectively. The temperatures of HCl and 2HCl loss obtained by TGA were 531 and 583 K, respectively, with a weight loss of 80% at ~900 K. The molecular weight sharply decreased between 520 and 650 K, with a corresponding weight loss of 70% at ~650 K. Subsequently, the crystals were analyzed using optical polarizing microscopy experiments with increasing temperature to investigate their thermal stability. The crystals were yellow at room temperature, as shown in the inset of Figure 2. As the temperature increased, the crystals changed from yellow to light brown and finally to dark brown, above 490 K, consistent with that shown in the XRD powder patterns of Figure 1. The possibility to change color was due to decomposition by loss of HCl and also due to the geometrical change of CuCl_4_. Near 540 K, the single-crystal surfaces exhibited slight melting. This temperature was similar to the temperature of HCl loss in the TGA experiment. Additionally, no endothermic peak corresponding to a phase transition above 200 K was observed in the differential scanning calorimetry (DSC) curve.

**Figure 3:**
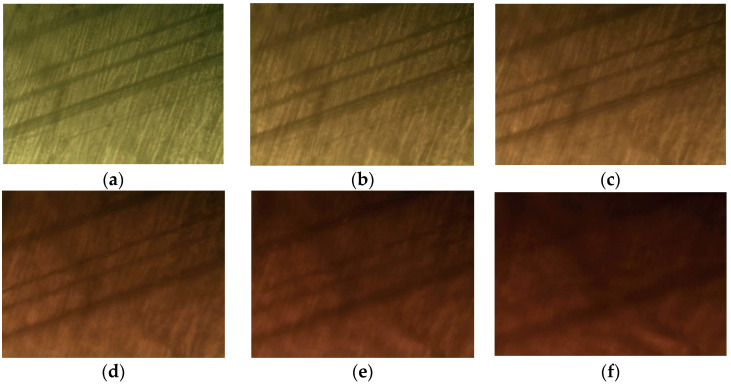
Optical polarizing microscopy images of [NH_3_(CH_2_)_5_NH_3_]CuCl_4_ at (**a**) 300 K, (**b**) 340 K, (**c**) 360 K, (**d**) 390 K, (**e**) 420 K, and (**f**) 440 K. Parallel lines represent ferroelastic twin domain walls.

A single crystal with ferroelastic properties has two or more orientation states, even in the absence of mechanical stress, and changes from one orientation state to another under mechanical stress [49,50]. The domain patterns observed under a polarized optical microscope are shown in Figure 3. One of the most common microstructures is related to twinning, with dominant twin planes oriented nearly perpendicular to each other. Ferroelastic domain patterns, represented by parallel lines, were observed at room temperature (Figure 3a). Although the crystal color changed with an increase in temperature, the twin domain patterns remained unchanged. Finally, the domain pattern turned dark brown near 440 K, as shown in Figure 3f, making it difficult to observe. The difficulty in observing the domain pattern above 440 K was due to the phenomenon in which single crystals begin to melt.

### 2.3. ^1^H NMR Chemical Shifts

**Figure 4:**
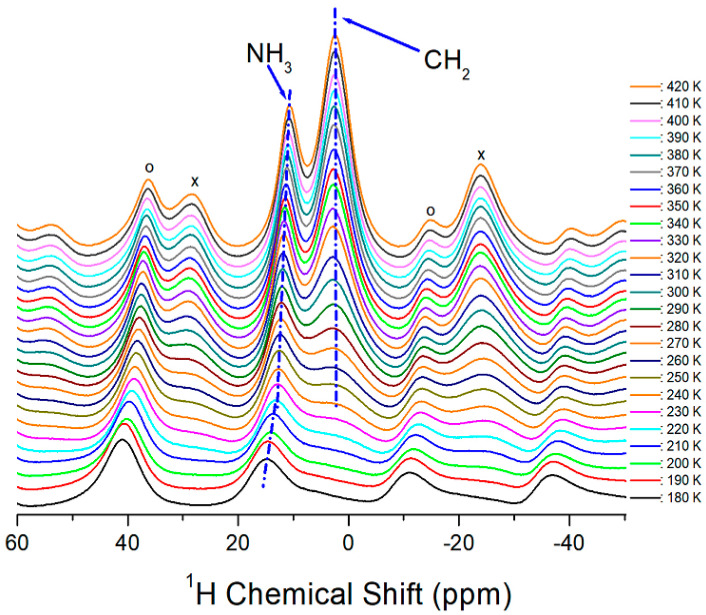
MAS ^1^H NMR spectra of [NH_3_(CH_2_)_5_NH_3_]CuCl_4_ as a function of temperature. Spinning sidebands are indicated by crosses and open circles.

The temperature dependence of the ^1^H MAS NMR spectra of the [NH_3_(CH_2_)_5_NH_3_]CuCl_4_ crystal was analyzed, and the ^1^H chemical shifts are shown in Figure 4. In the [NH_3_(CH_2_)_5_NH_3_] cation, the number of protons related to NH_3_ and CH_2_ was 6 and 10, respectively, and the intensity and linewidth of the ^1^H resonance peak were also related to the number of protons. The ^1^H signal in NH_3_ was observed at low temperatures, whereas the ^1^H signal in CH_2_ was difficult to observe, owing to its wide linewidth. Above 240 K, the NMR spectrum featured two resonance lines of NH_3_ and CH_2_. At 300 K, the ^1^H chemical shifts in NH_3_ and CH_2_ were 12.11 and 2.89 ppm, respectively. ^1^H signals for NH_3_ and CH_2_ overlap each other. Thus, their line widths could not be accurately distinguished in accordance with the temperature change; however, the line width of NH_3_ was narrower than that of CH_2_. The spinning sidebands for NH_3_ and CH_2_ are marked with open circles and crosses, respectively. The ^1^H chemical shifts of CH_2_, indicated by dotted lines in Figure 4, were almost independent of temperature.

The ^1^H chemical shift for NH_3_, from 180–220 K, was in the negative direction but shifted slightly in the negative direction at temperatures above that. Therefore, the structural environment of ^1^H in NH_3_ changed with the variation of temperature, while the environment of ^1^H in CH_2_ changed negligibly.

### 2.4. ^13^C NMR Chemical Shifts

**Figure 5:**
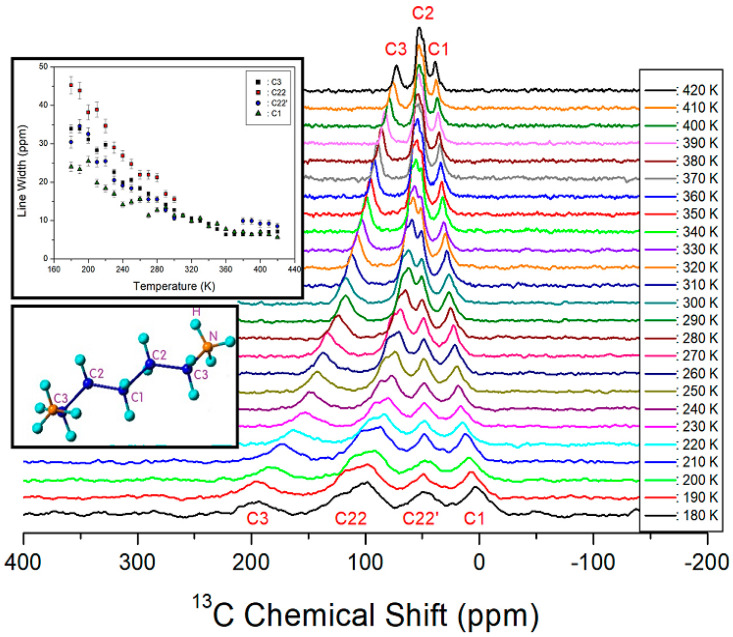
MAS ^13^C NMR spectra of [NH_3_(CH_2_)_5_NH_3_]CuCl_4_ as a function of temperature.

^13^C chemical shifts for the in-situ MAS NMR spectra with increasing temperature are shown in Figure 5. The tetramethylsilane (TMS) reference signal was recorded at 38.3 ppm at 300 K and considered to be the ^13^C chemical-shift standard. In the [NH_3_(CH_2_)_5_NH_3_] cation, the CH_2_ close to NH_3_ was labeled C3. The CH_2_ at the center of the cation was labeled C1, and the CH_2_ between C3 and C1 was labeled C2, as shown in the inset of Figure 5. At 300 K, the ^13^C chemical shifts were recorded at 27.19, 50.94, 62.95, and 118.46 ppm for C1, C22, C22′, and C3, respectively. The ^13^C chemical shifts for C1, C2, and C3, with temperature changes, are shown in Figure 5. The chemical shifts of C3 shifted rapidly in the negative direction with temperature change, while C1 shifted in a slightly positive direction. However, there were two different signals (C22 and C22′) for C2. Here, the chemical shift of C22 shifted in a negative direction, while that of C22′ shifted in a slightly positive direction, with a temperature change. The shifting of C22 and C22′ chemical shifts in different directions could be because of the position of C1 at the center of the cation and that of C2 between C1 and C3. In addition, at higher temperatures, the line widths for C1, C2, and C3, as shown in the inset of Figure 5, narrowed significantly owing to high internal mobility [51]. All ^13^C chemical shifts changed with the increase in temperature, with the C3 chemical shift exhibiting a rapid change.

### 2.5. ^1^H and ^13^C Spin-Lattice Relaxation Times

**Figure 6:**
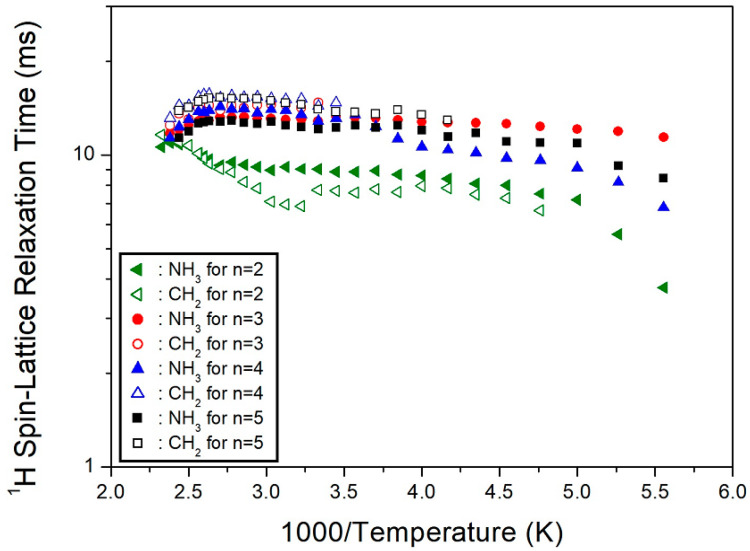
Temperature dependence of ^1^H NMR spin-lattice relaxation times (T_1ρ_) in [NH_3_(CH_2_)*_n_*NH_3_]CuCl_4_ (*n* = 2, 3, 4, and 5).

The intensities of the ^1^H MAS NMR and ^13^C MAS NMR spectra were measured by changing delay times at each temperature. The spectral intensity versus the delay time plot followed a mono-exponential function. The recovery traces of magnetization were characterized by the spin-lattice relaxation time, T_1ρ_, as [52,53,54]:

P_H(C)_(τ) = P_H(C)_(0)exp(−τ/T_1ρ_) (1)

where P_H(C)_(τ) and P_H(C)_(0) are signal intensities for the proton (carbon) at time τ and τ = 0, respectively. From the slope of the logarithm of intensity versus the delay time plot, the ^1^H T_1ρ_ values were determined for NH_3_ and CH_2_ at several temperatures. The intensity of each signal differed with the delay time. The results of ^1^H T_1ρ_ obtained here and the ^1^H T_1ρ_ of *n* = 2, 3, and 4 previously reported are shown in Figure 6 as a function of the inverse temperature. The ^1^H T_1ρ_ values were almost temperature independent and were in the order of 10 ms. However, the ^1^H T_1ρ_ values of NH_3_, represented with black squares, were shorter than those of CH_2_, marked with black open squares. Here, the T_1ρ_ values were compared according to the cation length from *n* = 2–5. The ^1^H T_1ρ_ values exhibited similar trends for different methylene chain lengths, with *n* = 2 exhibiting slightly shorter values than *n* = 3, 4, and 5.

**Figure 7:**
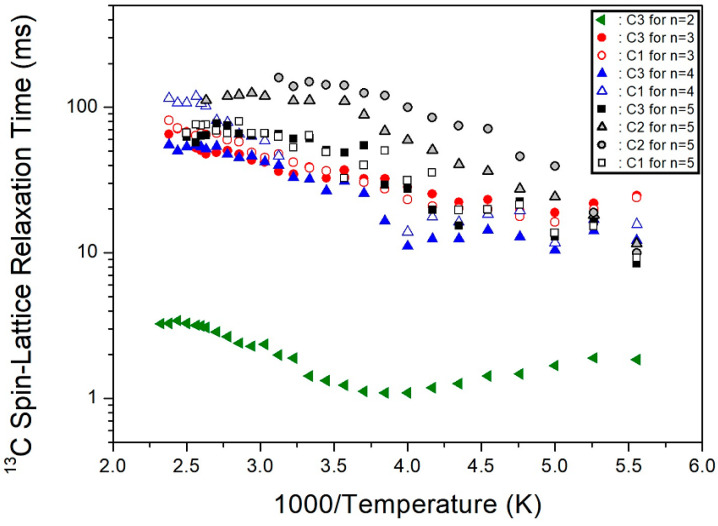
Temperature dependence of ^13^C NMR spin-lattice relaxation times (T_1ρ_) in [NH_3_(CH_2_)*_n_*NH_3_]CuCl_4_ (*n* = 2, 3, 4, and 5).

**Figure 8:**
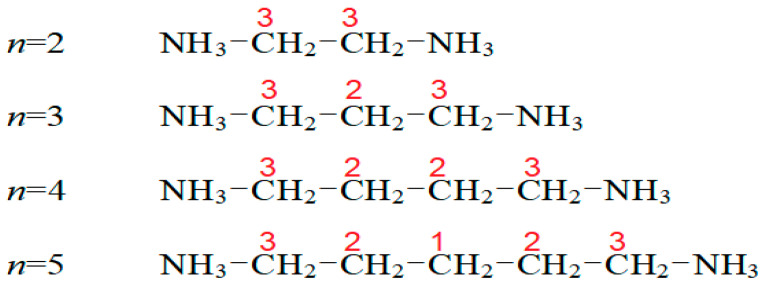
Names of carbons, according to their length, in the cation structure of [NH_3_(CH_2_)*_n_*NH_3_]CuCl_4._(*n* = 2, 3, 4, and 5).

The ^13^C T_1ρ_ values for C1, C2, and C3 were obtained as a function of the inverse temperature from the slope of the logarithm of intensity versus the delay time plot (Figure 7). The ^13^C T_1ρ_ values increased rapidly from 10–100 ms. The T_1ρ_ behavior for random motions, with a correlation time τ_C_, could be elucidated by a fast motion. The T_1ρ_ value of C3, located close to the paramagnetic Cu^2+^ ion, was shorter than that of C2, located further away from Cu^2+^. Additionally, the T_1ρ_ of C1, at the center of 5 CH_2_, exhibited very short values. It is interesting to compare the results for ^13^C T_1ρ_ according to the alkyl chain lengths. In the [NH_3_(CH_2_)*_n_*NH_3_] cation, the marks of C1, C2, and C3 along the length of n are shown in Figure 8. The ^13^C T_1ρ_ values exhibited similar trends for *n* =3, 4, and 5, with a very short value for *n* = 2, as shown in Figure 7. In the case of *n* = 5, unlike *n* = 2, 3, and 4, the T_1ρ_ value of C2 was different from those of C1 and C3. Overall, energy transfer was easier for the short alkyl chain length (*n* = 2).

## 3. Discussion

**Figure 9:**
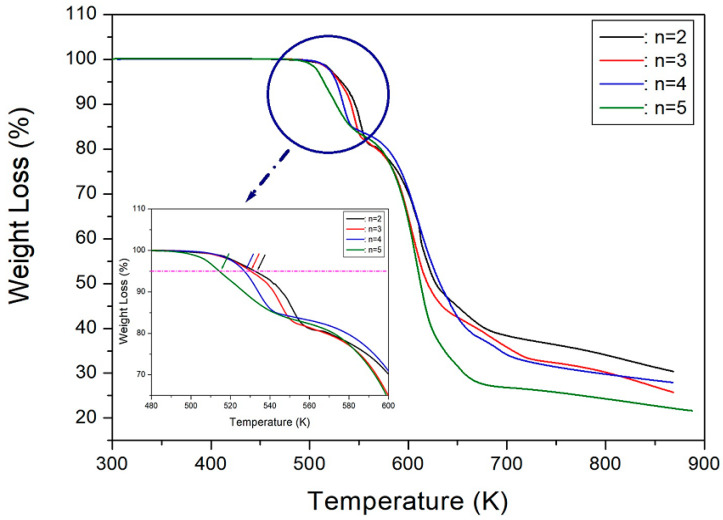
TGA curves of [NH_3_(CH_2_)*_n_*NH_3_]CuCl_4_ (*n* = 2, 3, 4, and 5) (inset: expansion of TGA curves near T_d_).

The thermal properties and structural dynamics of the [NH_3_(CH_2_)_*n*_NH_3_] cation in [NH_3_(CH_2_)*_n_*NH_3_]CuCl_4_ (*n* = 2, 3, 4, and 5) crystals were analyzed and compared using information obtained from TGA and NMR experiments. Thermal decomposition temperatures (T_d_) decreased with an increase in the value of *n*, as observed in the TGA results of the four crystals. An enlarged view was observed near T_d_; for *n* = 2, 3, 4, and 5, T_d_ values, when the case of 5% weight loss was set as T_d_, were 533, 530, 527, and 514 K, respectively, indicating no improvement in thermal stability with an increase in the cation length (Figure 9). The ^1^H and ^13^C T_1ρ_ values exhibited a similar trend in increasing the methylene chain length, with *n* = 2 exhibiting shorter T_1ρ_ values than *n* = 3, 4, and 5; T_1ρ_ increased with the increasing length of the CH_2_ chain, indicating that the energy transfer was not easy. The difference in T_1ρ_ values was mainly attributed to the cation length, with the shorter (*n* = 2) length exhibiting a smaller value, owing to the presence of paramagnetic Cu^2+^ ions. ^1^H T_1ρ_ values are very short after the inclusion of paramagnetic ions. The Cu^2+^ ions in [NH_3_(CH_2_)_*n*_NH_3_]CuCl_4_, which are paramagnetic and bonded with the inorganic layer through N–H···Cl hydrogen bonds, directly affected the ^1^H environment. With respect to the 2D structure of solar cell materials, the applicability of organic-inorganic hybrid compounds can be confirmed more clearly by knowing the energy transfer for a molecular motion for the spin-lattice relaxation times T_1ρ_ along the length of a cation.

## 4. Materials and Methods

[NH_3_(CH_2_)_5_NH_3_]CuCl_4_ single crystals were grown by gradually evaporating an aqueous solution of NH_2_(CH_2_)_5_NH_2_∙2HCl (Aldrich, 98%) and CuCl_2_ (Aldrich, 97%) at a constant temperature of 300 K. The grown single crystals that were 3 × 3 × 1.5 mm in size exhibited a yellow color.

The XRD powder pattern experiments of the [NH_3_(CH_2_)_5_NH_3_]CuCl_4_ crystal at several temperatures were measured in the measuring 2θ of 5–60° using an XRD system equipped with a Mo-Kα radiation source. The lattice parameters at various temperatures were determined by single-crystal X-ray diffraction (XRD) at the Seoul Western Center of the Korea Basic Science Institute (KBSI). A crystal block was picked up with paratone oil and mounted on a Bruker D8 Venture PHOTON III M14 diffractometer equipped with a graphite-monochromated Mo-Kα radiation source. Data were collected and integrated using SMART APEX3 (Bruker, 2016) and SAINT (Bruker, 2016). The absorption was corrected by a multi-scan method implemented in SADABS. The structure was solved using direct methods and refined by full-matrix least-squares on *F*^2^ using SHELXTL. All non-hydrogen atoms were refined anisotropically, and the hydrogen atoms were added to their geometrically ideal positions.

TGA and DTA experiments were performed in the temperature range of 300–873 K on a thermogravimetric analyzer (TA Instruments) at a heating rate of 10 K/min with an N_2_ gas flow [42]. Additionally, a twin domain pattern, observed in the 300–680 K temperature range, was measured using an optical polarizing microscope by placing the prepared single crystals on a Linkam THM-600 heating stage.

NMR chemical shifts and spin-lattice relaxation times (T_1ρ_) for ^1^H and ^13^C in [NH_3_(CH_2_)_5_NH_3_]CuCl_4_ crystals were measured using a Bruker 400 MHz Avance II+ solid-state NMR spectrometer at the same facility, KBSI. The Larmor frequency was ω_o_/2π = 400.13 MHz for ^1^H NMR, and ω_o_/2π = 100.61 MHz for ^13^C NMR. To minimize the spinning sideband, the sample tube spinning speed was set to 10 kHz, and TMS was used as reference material to accurately measure the NMR chemical shifts. T_1ρ_ values were obtained using a π/2−τ pulse, followed by a spin-lock pulse of duration τ, and the width of the π/2 pulse for ^1^H and ^13^C was in the 3.2–3.9 μs range. The temperature was changed by adjusting the N_2_ gas flow and the heater current, and the NMR experiment was conducted in the 180–430 K temperature range.

## 5. Conclusions

We discussed XRD, TGA, and NMR experiments to investigate the crystal structure, thermal stabilities, and physical properties of [NH_3_(CH_2_)_5_NH_3_]CuCl_4_ crystal. First, the monoclinic structure and lattice parameter were confirmed by XRD, and its thermodynamic property was observed at about 514 K without phase transition. NMR analysis indicated that the crystallographic environment of ^1^H in NH_3_ and that of ^13^C on C3, located close to NH_3_ at both ends of the cation, were changed, indicating a large structural change of CuCl_4_ connected to the N–H···Cl. The effects of the length of CH_2_ in the cation on the molecular motions and thermal properties will facilitate future research on their potential application in the research of environment-friendly hybrid perovskite solar cells.

## Data Availability

Not applicable.

## Sample Availability

Samples of the compounds are available from the authors.
